# Microbial spectrum of cervicovaginal discharge in symptomatic Indian women of reproductive age group

**DOI:** 10.6026/973206300210853

**Published:** 2025-04-30

**Authors:** Neelima Singh, Dipali Prasad, Kumar Saurabh

**Affiliations:** 1 Department of Microbiology, Himalaya Medical College and Hospital, Patna, Bihar, India; 2 Department of Obstetrics and Gynaecology, IGIMS, Patna, Bihar, India; 3 Department of Microbiology, IGIMS, Patna, Bihar, India

**Keywords:** Cervicovaginal discharge, bacterial vaginosis, aerobic vaginitis, vulvovaginal candidiasis, *Trichomonas vaginalis*, microbial spectrum, reproductive health

## Abstract

Cervicovaginal discharge is commonly caused by infections like *aerobic vaginitis (AV)*, *bacterial vaginosis (BV)*, *vulvovaginal candidiasis (VVC)* and *trichomoniasis*. Microbial growth was found in 77.27% of samples, with AV being the most prevalent (35%) in a study of 220 symptomatic women. Bacterial vaginosis was confirmed using Nugent's and Amsel's criteria. *Enterococcus spp.*, *E. coli* and *Staphylococcus aureus* were major AV pathogens. *Non-albicans Candida species* were more common than *C. albicans* in VVC cases. Hence, accurate microbial identification is vital for targeted therapy, especially given rising non-albicans Candida prevalence.

## Background:

Vaginal discharge is a common gynecological complaint among women of reproductive age and can be either physiological or pathological. Normal vaginal secretions are typically white and floccular, collecting in the posterior fornix, the most dependent part of the vagina [1]. Pathological vaginal discharge, often indicative of infections such as vaginitis or cervicitis, can present with symptoms ranging from increased discharge, malodor, pruritus and dyspareunia to lower abdominal pain [2]. Importantly, cervicitis may be asymptomatic or present with mild symptoms, leading to undiagnosed and untreated infections that contribute to reproductive morbidities [3]. The vaginal microbiota plays a crucial role in maintaining vaginal health. It is primarily dominated by Lactobacillus species, which help in maintaining an acidic pH (<4.5) through lactic acid production, preventing colonization by pathogenic organisms [4]. However, disturbances in this microbiota can lead to infections such as *bacterial vaginosis (BV)*, *vulvovaginal candidiasis (VVC)*, *trichomoniasis* and *aerobic vaginitis (AV)* [4]. Vaginitis, an inflammatory condition of the vaginal mucosa, is a prevalent issue in gynecological practice and accounts for a significant number of outpatient visits worldwide [6]. The three most common etiologies of vaginitis are BV, VVC and *trichomoniasis*, with bacterial vaginosis being the most frequently diagnosed, accounting for 40-50% of cases [7, 8]. The prevalence of bacterial vaginosis varies globally, with an estimated 90 million cases reported annually [9]. In India, its prevalence ranges from 17.8% to 63.7% [10]. *aerobic vaginitis (AV)*, a relatively newer classification of vaginal infection, is characterized by a shift in the vaginal flora, replacing lactobacilli with facultative aerobic pathogens such as *Escherichia coli*, *Staphylococcus aureus*, *Enterococcus spp.*, *Klebsiella pneumoniae* and Group B *Streptococcus* [11]. Unlike BV, which is associated with an overgrowth of anaerobes, AV is an inflammatory condition marked by increased vaginal pH (>6), reduced lactate concentration and increased leukocyte and pro-inflammatory cytokine levels [12]. It presents with symptoms such as yellowish vaginal discharge, dyspareunia and vaginal inflammation [13].

Cervicitis, another common gynecological condition, involves inflammation of the endocervix, which can be acute or chronic. Acute cervicitis is often caused by sexually transmitted infections (STIs) such as *Neisseria gonorrhoeae* and Chlamydia trachomatis, while chronic cervicitis may result from long-term irritation or secondary infections [14]. Bacterial vaginosis is a complex condition involving the loss of lactobacilli and overgrowth of organisms such as Gardnerella vaginalis, *Mobiluncus spp*., *Prevotella spp.*, *Porphyromonas spp.*, *Bacteroides spp.* and *Mycoplasma hominis* [15]. It is diagnosed based on Amsel's clinical criteria or Nugent's scoring system, with characteristic features including increased vaginal pH, presence of clue cells, thin homogenous discharge and a positive whiff test [16]. Vulvovaginal candidiasis, caused by an overgrowth of Candida species, is a significant cause of vaginal infections, affecting up to 40% of women presenting with vaginal complaints [17]. It is characterized by thick curd-like discharge, itching and erythema. *Candida albicans* is the most common causative species; however, *Non-albicans Candida species*, such as *C. glabrata*, *C. tropicalis* and *C. parapsilosis*, are emerging as significant pathogens, often exhibiting resistance to standard antifungal treatments [18]. *trichomoniasis*, caused by the protozoan parasite *Trichomonas vaginalis*, is the most common non-viral sexually transmitted infection globally, affecting approximately 180 million women annually [19]. It is associated with adverse reproductive outcomes such as preterm birth, low birth weight, increased susceptibility to HIV infection and potential links to cervical and prostate cancers [20]. Therefore, it is of interest to investigate the microbial spectrum of cervicovaginal discharge in symptomatic women attending the gynecology outpatient department at Indira Gandhi Institute of Medical Sciences (IGIMS), Patna.

## Materials and Methods:

This hospital-based cross-sectional study was conducted in the Gynaecology Outpatient Department (OPD) of Indira Gandhi Institute of Medical Sciences (IGIMS), Patna, over a period of one year from December 2016 to December 2017. Ethical approval was obtained from the Institutional Ethics Committee and informed written consent was secured from all participants after explaining the study's objectives and procedures. The study included non-pregnant women aged 18-45 years who presented with complaints of vaginal discharge. Women who were pregnant, in the puerperium, within six weeks post-abortion, experiencing menstrual bleeding or those with a confirmed sexually transmitted infection (STI) were excluded. Additional exclusion criteria included recent antibiotic or antifungal use, prior hysterectomy and inability to undergo pelvic examination. A total of 660 samples were collected from 220 patients, consisting of high vaginal swabs and cervical swabs. The high vaginal swabs were collected using sterile swabs inserted into the upper vaginal region to avoid contamination, with three swabs per patient designated for pH testing, Gram staining and culture. Cervical swabs were obtained using a sterile speculum, clearing cervical mucus before sample collection to ensure accurate microbiological analysis. In the laboratory, vaginal secretion pH was measured using indicator strips, while the Whiff test was performed by adding 10% potassium hydroxide (KOH) to assess the presence of a characteristic "fishy odor". Microscopy was performed through wet mount and KOH mount preparations to detect pus cells, *Trichomonas vaginalis*, clue cells and yeast-like organisms.

Gram staining was conducted for bacterial morphology assessment, with the Nugent scoring system applied for bacterial vaginosis diagnosis. Additionally, Amsel's criteria were used to confirm bacterial vaginosis if at least three of the following were present: vaginal pH >4.5, homogeneous thin discharge, a positive Whiff test, or clue cells on saline microscopy. The severity of *aerobic vaginitis (AV)* was determined using Donders' scoring system, classifying cases as mild, moderate, or severe based on the presence of inflammatory changes and disruption of vaginal flora. Culture and identification of microorganisms were carried out using a variety of media to isolate bacterial and fungal pathogens. Blood agar and MacConkey agar were used for differentiating aerobic bacterial species, while chocolate agar facilitated the isolation of fastidious organisms like *Neisseria gonorrhoeae*. Fungal cultures were grown on Sabouraud's Dextrose Agar (SDA) with chloramphenicol, Corn Meal Agar with Tween 80 and CHROM Agar, which enabled species differentiation based on colony morphology and pigmentation. Biochemical tests, including catalase and coagulase tests for Staphylococcus species, bile esculin test for *Enterococcus species*, oxidase and indole tests for *Pseudomonas aeruginosa* and *Escherichia coli*, citrate utilization test for enteric bacteria and the urease test for *Klebsiella pneumoniae*, were performed for bacterial identification. The Triple Sugar Iron (TSI) test was also employed to differentiate enteric bacteria based on carbohydrate fermentation and hydrogen sulfide production. Identification of Candida species was further confirmed using the Germ Tube Test, chlamydospore formation on Corn Meal Agar and color differentiation on CHROM Agar. Statistical analysis was conducted using Microsoft Excel, with categorical variables expressed as percentages and frequencies. The results were represented in tables and graphical formats to illustrate trends in microbial distribution among symptomatic patients.

## Results:

A total of 220 patients with symptomatic cervicovaginal discharge were included in the study, of which 170 (77.27%) cases yielded bacterial and fungal isolates, while 50 cases (22.73%) showed no growth. Among the positive cases, the most prevalent infection was *aerobic vaginitis (AV)* in 77 cases (35%), followed by *bacterial vaginosis (BV)* in 61 cases (27.72%), *vulvovaginal candidiasis (VVC)* in 24 cases (10.9%) and *trichomoniasis* in 8 cases (3.63%) (Table 1). The majority of patients presented with pain in the lower abdomen and increased purulent, offensive vaginal discharge (150 cases, 68.19%). Other symptoms included pain in the lower abdomen with increased non-purulent discharge (40 cases, 18.19%) and pruritus with thick curdy discharge (30 cases, 13.63%) (Table 2). Among 77 cases of Aerobic Vaginitis, the most frequently isolated pathogens were *Enterococcus species* (20 cases, 25.97%), *Escherichia coli* (18 cases, 23.37%), *Staphylococcus aureus* (15 cases, 19.48%) and Coagulase-negative Staphylococci (10 cases, 12.98%). Other organisms isolated included *Klebsiella pneumoniae* (4 cases, 5.19%), *Pseudomonas aeruginosa* (3 cases, 3.89%), *Acinetobacter species* (3 cases, 3.89%) and mixed growth (4 cases, 5.19%) (Table 3). Among 24 cases of Vulvovaginal Candidiasis, *Non-albicans Candida species* (18 cases, 75%) were more prevalent than *Candida albicans* (6 cases, 25%). Species differentiation using CHROM agar showed that *C. tropicalis* (9 cases, 37.5%) was the most commonly isolated *Non-albicans Candida species*, followed by *C. glabrata* (5 cases, 20.83%) and *C. parapsilosis* (4 cases, 16.7%). *Candida albicans* was confirmed in 6 cases (25%) through germ tube tests and light green colony identification on CHROM agar (Table 4). These findings highlight the microbial diversity in cervicovaginal discharge cases, with Aerobic Vaginitis being the predominant infection followed by Bacterial Vaginosis. Candida infections showed a shift towards non-albicans species, emphasizing the need for accurate microbial diagnosis to ensure appropriate treatment.

## Discussion:

The study demonstrated that 77.27% of symptomatic women had microbial isolates, with the highest prevalence attributed to AV (35%) and followed by bacterial vaginosis (27.72%), VVC (10.9%) and TV (3.63%). The presence of a significant number of women (22.73%) with no detectable microbial growth highlights the possibility of non-infectious causes such as hormonal imbalances or atrophic vaginitis [3]. A majority of women presented with complaints of increased purulent and offensive vaginal discharge (68.19%), followed by pain in the lower abdomen and pruritus. These findings are consistent with previous studies that reported vaginal discharge as the most common symptom of reproductive tract infections [5, 6]. Poor personal hygiene was observed in 87.27% of cases, underscoring its role as a significant risk factor for vaginal infections, as corroborated by Jindal *et al.* who reported similar findings [7]. *trichomoniasis*, a prevalent yet often overlooked STD, poses significant public health risks and faces growing concerns over metronidazole resistance [8, 9]. AV was the most frequently diagnosed condition, with *Enterococcus species* (25.97%) being the predominant pathogen, followed by *E. coli* (23.37%) and *Staphylococcus aureus* (19.48%). These findings align with previous studies where Enterococcus faecalis and *E. coli* were the primary aerobic pathogens in AV [10]. The predominance of Gram-positive organisms in AV (58.44%) suggests a shift from lactobacilli dominance to a dysbiotic, inflammatory vaginal environment [11].

Bacterial vaginosis was diagnosed using both Nugent's scoring and Amsel's criteria, with Nugent's scoring identifying 27.72% of cases. Bacterial vaginosis is known to be a polymicrobial condition characterized by a loss of Lactobacillus species and an overgrowth of anaerobic bacteria such as Gardnerella vaginalis and *Mobiluncus spp*. [12]. The prevalence of bacterial vaginosis in this study is comparable to other Indian studies reporting rates between 25% and 40% [13]. The strong association between bacterial vaginosis and STIs underscores its importance in reproductive health [14]. Among the 10.9% of women diagnosed with candidiasis, non-albicans species (75%) were more prevalent than *Candida albicans* (25%). The increasing incidence of non-albicans Candida, particularly *C. glabrata* (20.83%) and *C. tropicalis* (37.5%), suggests a shift in the epidemiology of VVC, which has been reported in multiple studies [15]. The prevalence rates align with other studies, such as that by Donders *et al.* which emphasized AV as a common but often overlooked condition [15]. This shift is of clinical importance as non-albicans species exhibit increased resistance to conventional azole therapy [16]. TV was diagnosed in only 3.63% of cases using wet mount microscopy, a method known to have limited sensitivity compared to culture or molecular diagnostics [17]. The prevalence of TV varies geographically, with higher rates reported in sexually active populations. The association of TV with bacterial vaginosis, as observed in 62.5% of cases, has been well-documented due to shared risk factors and disruption of vaginal flora [18]. Bacterial vaginosis was found to coexist with candidiasis (13.11%) and *trichomoniasis* (4.91%), highlighting the polymicrobial nature of vaginal infections [19]. Studies have indicated that bacterial vaginosis associated dysbiosis predisposes the vaginal environment to secondary infections, increasing susceptibility to HIV and other STIs [20].

## Conclusion:

AV and bacterial vaginosis are the predominant causes of abnormal vaginal discharge in symptomatic Indian women. The increasing prevalence of non-albicans Candida and the association of bacterial vaginosis with other infections emphasize the need for comprehensive diagnostic and treatment approaches. Routine microbiological evaluation, along with patient education on hygiene and STI prevention, can significantly reduce the burden of vaginal infections in reproductive-aged women.

## Figures and Tables

**Table 1:** Distribution of causes of abnormal vaginal flora

**Infection Type**	**Number of Cases (n=220)**	**Percentage (%)**
Aerobic Vaginitis	77	35%
Bacterial Vaginosis	61	27.72%
Candidiasis	24	10.90%
Trichomoniasis	8	3.63%
Normal (No Growth)	50	22.73%

**Table 2:** Distribution of patients based on clinical presentation

**Complaints**	**Number of Cases (n=220)**	**Percentage (%)**
Pain L/A + Increased purulent and offensive discharge	150	68.19%
Pain L/A + Increased non-purulent discharge	40	18.19%
Pruritus with thick curdy discharge	30	13.63%

**Table 3:** Distribution of microorganisms in aerobic vaginitis cases

**Organism Isolated**	**Number of Cases (n=77)**	**Percentage (%)**
*Enterococcus species*	20	25.97%
*Escherichia coli*	18	23.37%
*Staphylococcus aureus*	15	19.48%
*Coagulase-negative Staphylococci (CONS)*	10	12.98%
*Klebsiella pneumoniae*	4	5.19%
*Pseudomonas aeruginosa*	3	3.89%
*Acinetobacter species*	3	3.89%
Mixed growth	4	5.19%

**Table 4:** Candida species distribution based on CHROM Agar

**Candida Species**	**Number of Cases (n=24)**	**Percentage (%)**
*C. albicans*	6	25%
*C. tropicalis*	9	37.50%
*C. glabrata*	5	20.83%
*C. parapsilosis*	4	16.70%
